# Lymphocyte subpopulation in healthy and diseased gingival tissue

**DOI:** 10.4103/0972-124X.44091

**Published:** 2008

**Authors:** Aniz Amunulla, Remya Venkatesan, Hemalatha Ramakrishnan, K. V. Arun, Subitha Sudarshan, Avaneendra Talwar

**Affiliations:** 1 *Lecturer, KIDS, India*; 2 *Professor, Department of Periodontics, Ragas Dental College and Hospital, Chennai, India*; 3 *Professor, Department of Periodontics, Ragas Dental College and Hospital, Chennai, India*; 4 *Professor, Department of Periodontics, Ragas Dental College and Hospital, Chennai, India*; 5 *Professor and Head, Department of Periodontics, Ragas Dental College and Hospital, Chennai, India*; 6 *Lecturer, Department of Periodontics, Ragas Dental College and Hospital, Chennai, India*

**Keywords:** Lymphocytes, periodontal disease, immunohistochemistry

## Abstract

In this study, infiltrating lymphocytes subpopulation in gingival sections of healthy, inflamed, and periodontitis lesions was investigated. A set of cluster of differentiation (CD) antigen specific monoclonal/polyclonal antibodies to detect different cell types within the tissues was used. These included anti-CD3 (pan T-cell), anti-CD45RO (memory T-cell), anti-CD20 (B-cell), and kappa light chain (plasma cells). Biopsies of gingival tissue were obtained from 17 patients who had clinically healthy gingiva, from 18 patients with gingivitis, and 17 patients with periodontitis.

A significantly greater proportion of T-cells (*P* < 0.00) was observed in healthy gingival and gingivitis tissue samples compared to periodontitis tissue samples. In addition, a greater proportion of B-cells was observed in periodontitis lesions than in the gingival lesions (*P* < 0.00). The memory T-cells and the kappa light-chain plasma cells were present in both healthy and diseased tissues, suggestive of previous activation by periodontal pathogenic microorganisms.

In conclusion, these differences in the relative proportions of B- and T-cells may reflect a difference in the immunopathology of periodontitis and gingivitis lesions.

## INTRODUCTION

Periodontitis is an infection of microbial origin, the pathogenesis of which is mediated through the host immune response. The immune response to the bacteria can minimize the damage to periodontal tissues by eliminating the pathogens or neutralizing their products. There is also evidence to suggest that much of the hard and soft tissue destruction is mediated by elements of the immune response. Hence, the host response is believed to have both a protective, as well as a destructive role in periodontitis.[1]

It is the nature of the inflammatory response, which determines the destructive character of periodontal disease. With increased bacterial challenge, bacterial products interact with the gingival epithelium to induce the expression of adhesion molecules and the production of proinflammatory cytokines and chemokines, which guide the leukocytes into the gingival tissue and ultimately into the gingival sulcus through the junctional epithelium. However, with continued presence of bacteria, the formation of an inflammatory infiltrate in the connective tissue consisting predominantly of lymphocytes (T-cells) and macrophages take place. Subsequently, in a susceptible person, this adaptive response still does not contain the microbial challenge leading to a change in nature of the inflammatory response (B-cells/plasma cells) which results in either protective antibodies and subsequent control of infection or nonprotective antibodies and connective tissue destruction and bone loss.[2]

Both T- and B- lymphocytes are present in sites of healthy and periodontitis tissues and are considered to play an essential role in the pathogenesis of the disease.[3] The central role played by T-cells in mediating the host response against periodontopathogens in periodontal disease has been well recognized. They regulate polyclonal B-cell activation as well as antigen-specific antibody formation.[4] Naive T-cells (CD3) on their own are unable to recognize microbial antigens efficiently. Efficient T-cell response is dependent on the presence of specialized group of cells called the antigen presenting cells (APCs). These APCs recognize pathogen associated molecular patterns (PAMPs) produced by microorganisms and present them as peptides to the naive T-cell in a manner that allows them to mount an effective response that clears the bacterial antigen (Ag). The exposure of the naive T-cell to microbial antigens results in the synthesis of new proteins, cellular proliferation, and differentiation of the naive T-cell into effector and memory T-cells (CD45RO), which is specific for the activating antigen. Majority of T-cells in periodontitis lesions express CD45RO.[5,6]

Previous studies by Yamazaki and Nakajima *et al*, reported that there was a higher proportion of CD45RO in gingivitis and periodontitis than CD45RA naive cells.[6,7] However, the proportion of memory T-cells present in total T-cell infiltrate is yet to be fully characterized.

B-cells (CD20) are professional APCs which can recognize PAMPs, but require contact with memory T–cells to differentiate into plasma cells, which then, generate immunoglobulin that facilitate antigen opsonization and phagocytosis. The immunoglobulin, produced in response to periodontopathogens, result in a wide diversity due to polyclonal activation of the B-cell. Thus, the immunoglobulin produced may or may not be protective.[8]

It has been suggested that T-cells dominate the gingivitis lesion, with an increase in the number of B-cells in the periodontitis lesion,[9] albeit under T-cell control.[10]

The literature reports on the relative proportion of lymphocytes in the gingival tissue and their effect on the periodontal tissue are contradictory and do not support a defined answer on the matter.[11,12]

The present study was undertaken to determine the relative proportion of lymphocytes infiltrate in healthy gingival, gingivitis, and periodontitis tissues.

## MATERIALS AND METHODS

### Subject selection

Fifty two patients were randomly selected from the patient pool attending the out-patient clinic, Department of Periodontics, Ragas Dental College and Hospitals, Chennai, and were enrolled in the study between January and August 2007. Patients exhibiting good general health with no history of smoking or periodontal treatment or antimicrobial therapy for the past six months were chosen for the study. The patients were divided into three groups: group A consisted of 17 patients with clinically healthy gingiva (probing depth (PD) ≤ 3 mm, no clinical attachment loss (CAL), and no bleeding on probing (BOP); group B comprised of 18 patients with gingivitis (PD ≤ 3 mm, BOP, no CAL); and group C comprised of 17 patients with periodontitis (PPD ≥ 5 mm and CAL ≥ 3 mm in at least six sites). The Institutional Review Board of Ragas Dental College and Hospitals approved the study protocol and a written informed consent was obtained from each subject.

### Tissue collection

Gingival samples were collected from group A and B patients while they underwent extraction for orthodontic reasons or during third molar removal and from group C patients during surgical periodontal therapy. The tissues obtained from the patients were fixed with 10% neutral buffered formalin and then paraffin embedded within 48 hours, to avoid degradation of the antigens. Four-microns thick sections were made from the specimens and mounted on 3-aminopropyltriethoxysilane (APES) coated slides.

### Antibodies used for immunohistochemistry

The following primary antibodies were used for immunohistochemistry:

- Monoclonal mouse antihuman CD3 (Code no. N 0756, Dako, Denmark) reacts with ε-chain of CD3 part of the TCR/CD3 complex. This antibody is a pan T-cell reagent for the detection of normal T-cells.
- Monoclonal mouse anti-human CD45RO (Code no. N1525, Dako, Denmark) reacts with an epitope of CD45RO. The antibody labels mature and activated T-cells of CD4 and CD8 subsets.
- Monoclonal mouse anti-human CD20 (Code no. N15020, 0Dako, Denmark) reacts with an epitope located on the surface of the B-cell. The antigen designated CD20 appears early during B-cell maturation and is lost shortly before the terminal plasma-cell stage. CD20 is confined to B-cells.
- Polyclonal rabbit antihuman kappa light chain (Code no. N1510, Dako, Denmark) labels specifically for kappa-positive plasma cells. Activated B-cell or plasma cell produces immunoglobulins, which consists of two heavy chains and two light chains on their surface for recognition of the microbial antigen. The heavy chains are specific to the immunoglobulin type, while the light chain kappa (κ) and lambda (λ) are common. It has been shown that antibodies produced against periodontal pathogens have a predominance of κ light chains.[13] Hence, κ light chain was chosen as a marker for activated B-cell or plasma cell.

### Immunohistochemisty method

The tissues were deparaffinized, transferred to citrate buffer, and autoclaved for antigen retrieval at 15 lbs pressure for 15 minutes. The slides were then dipped in two changes of phosphate buffered saline (PBS) for 5 minutes and treated with 3% hydrogen peroxide for 10 minutes to quench endogenous peroxidase activity. The slides were put in two changes of PBS and then treated with power block for 20 minutes.

The primary antibody protein was added to the tissue on the slides. The petridish containing the slides was kept at room temperature for 1 hour. The sections were washed in two changes of cold PBS for 10 minutes each and a drop of super enhancer and incubated for 30 minutes. A drop of poly horseradish peroxide (HRP) was then added onto the sections and incubated for 30 minutes. The sections were then washed in two changes of cold PBS for 10 minutes each. A drop of freshly prepared 3′ – Diamino benzidine (DAB) tetra hydrochloride – a substrate chromogen – was added onto the sections. Slides were then washed in running distilled water and counter stained with Harris's Hematoxylin. The tissue sections were washed with acid alcohol and xylene and mounted with DPX.

The procedure outlined above was repeated for studying CD3, CD45RO, CD20, and κ light chain expression on different tissue sections. All the slides were observed under the microscope at magnification ×10 and ×40. Throughout the procedure, care was taken not to dry the tissues. The slides were checked for positive staining.

### Statistical evaluation

Analysis of CD3, CD45RO, CD20, and κ light chain expression was done by evaluating the labeling index (LI). The percentage positive cells was the ratio between the number of cells that showed intense staining divided by the total number of cells counted. A total of thousand cells was counted in each slide.

$$\mathrm{LI}\;=\;\frac{\mathrm{Number of positive cells}}{1000}\;\times \;100$$

Data entry and descriptive analysis was performed using SPSS version 10.0.5^®^. Analysis of variance (ANOVA) followed by Bonferroni test was used to compare the groups, healthy, gingivitis, and periodontitis with regard to the CD3, CD45RO, CD20, and κ light chain antibody.

## RESULTS

**Graph 1:**
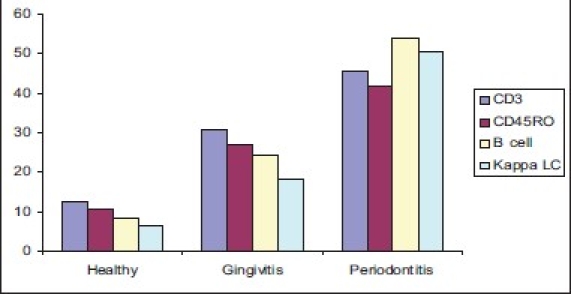
Labeling index of CD3, CD45RO, CD20, kappa light chain in healthy, gingivitis and periodontitis groups

The present study was carried out to investigate relative proportion of infiltrated naive T-cells, memory T-cells, B-cells, and κ-positive plasma cells in the connective tissue of gingival sections taken from patients with clinically healthy gingiva, gingivitis, and periodontitis. A set of CD antigen specific monoclonal/polyclonal antibodies to detect different cell types within the tissues was used. These included anti-CD3 (pan T- cell), anti-CD45RO (memory T-cell), anti-CD20 (B-cell), and κ light chain (plasma cells). The entire tissue sample examined showed both the epithelium and connective tissues. No staining was observed in the epithelium in any of the sections. Table 1 and Graph 1 summarizes the results of this study.

**Table 1:** Labeling index and ratio of naïve and activated lymphocytes in healthy, gingivitis and periodontitis groups

Groups	Labeling index (LI) (mean ± standard deviation)		CD45RO:CD3 ration	Labeling index (LI) (mean ± standard deviation)		CD20: κ-light chain ratio

	CD3	CD45RO		CD20	κ-light chain	
Healthy gingiva	12.25± 2.17	10.53± 1.35	85.959%	8.34± 0.16	6.27±0.32	75.179
Gingivitis	30.44± 3.64	26.78± 2.51	87.976%	24.21± 1.42	18.34±0.64	75.588
Periodontitis	45.35± 2.43	41.62± 1.37	91.775%	53.83± 1.68	50.23±2.27	93.256
p- value	0.00	0.00		0.00	0.00

P < 0.001 Statistically significant

### CD3

**Figure 1:**
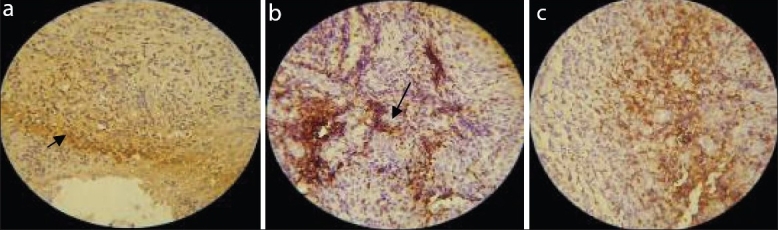
CD3 positive cells in healthy (a), gingivitis (b) and peridontitis (c) group. Arrow indicates CD3 positive cells. Magnifi cation 40×

The mean LI of CD3 antibody in the connective tissue layer of healthy gingival group was 12.25 ± 2.71, in gingivitis group it was 30.44 ± 3.64, and in the periodontitis group it was 45.35 ± 2.43. There was a statistically significant difference in the mean LI between all the three study groups (*P*= 0.00) [Figure 1].

### CD45RO

**Figure 2:**
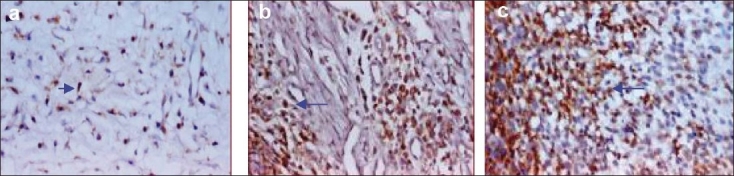
CD45RO positive cells in healthy (a), gingivitis (b) and peridontitis (c) group. Arrow indicates CD45RO positive cells. Magnification 40×

The mean LI of CD45RO [Figure 2] antibody was 10.53 ± 1.35 in the connective tissue layer of healthy gingival group, 26.78 ± 2.51 in the gingivitis group, and 41.62 ± 1.37 in the periodontitis group. There was a statistically significant difference in the mean LI between all the three study groups (*P* = 0.00).

### Memory T-cell to naive T-cell ratio - Proportion of CD45RO to CD3

The memory T-cell to naive T-cell ratio was obtained by dividing the mean LI of CD45RO by that of CD3. The memory T-cell to naive T-cell ratio was 85.96% in healthy gingival group, 87.98% in gingivitis group, and 91.76% in periodontitis group.

### CD20

**Figure 3:**
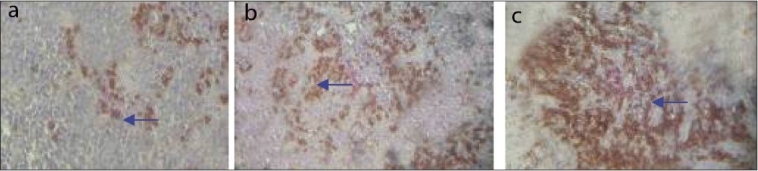
CD20 positive cells in healthy (a), gingivitis (b) and peridontitis (c) group. Arrow indicates CD20 positive cells. Magnification 40×

The B-cell [Figure 3] showed diffuse staining in all the layers of connective tissue in the healthy gingiva and gingivitis groups, whereas in the periodontitis group, the B-cells tended to be clumped together in clusters immediately subjacent to the epithelium.

**Figure 4:**
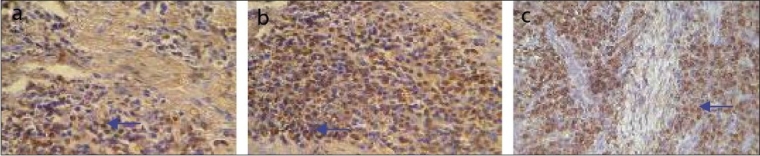
Kappa light chain positive cells in healthy (a), gingivitis (b) and peridontitis (c) group. Arrow indicates Kappa light chain positives cells. Magnification 40×

In the healthy gingival group the mean LI for B-cells was 8.34 ± 0.16, in the gingivitis group it was 24.21 ± 1.42, and in the periodontitis group it was 53.83 ± 1.68. There was a statistically significant difference in the mean LI between all the three study groups (*P* = 0.00).

### Kappa light chain

In healthy gingiva group the mean LI for κ-positive plasma cells was 6.27 ± 0.32, in gingivitis group it was 18.34 ± 0.64, and in periodontitis group it was 50.23 ± 2.71.There was a statistically significant difference (*P* = 0.000**) between all the three groups.

### Proportion of plasma cell to B-cell

The plasma cell to B-cell ratio was obtained by dividing the mean LI of CD20 by that of κ light chain, which is an indicator of the B-cell activation within the gingival tissue.

The plasma cell to B-cell ratio was 75.18% in healthy gingiva, 75.59% in gingivitis group, and 93.25% in periodontitis group. There was a statistically significant difference between the periodontitis group and healthy gingiva group (*P* = 0.00) and between gingivitis and healthy gingiva groups (*P* = 0.00). There was however no statistically significant difference between periodontitis and gingivitis groups.

## DISCUSSION

It has been suggested that immune response to bacterial aggression plays an important role in periodontal disease.[14] The innate immune system in the periodontium consists of several cells such as epithelial cells, Langerhans cells, tissue macrophages, neutrophils, and dentritic cells which directly or indirectly participate in antigen presentation to prime B- and T-cells that constitute the mucosal adaptive immune response, which is involved in the complete elimination of the pathogens.[15]

Several lines of evidence suggest that periodontal diseases may result from or cause imbalance in the regulation of the local immune response. This dysfunction may differentially affect T- and B-lymphocyte proportion in the periodontal tissue.[16,17] Conflicting results have been obtained by studies which have focused on whether the conversion from gingivitis to periodontitis is associated with distinction phenotype of lymphocyte infiltrate.[18–21]

There is as yet no consensus in the exact role played by the adaptive immune cells in mediating the pathogenic processes in periodontal disease. The purpose of the present study was to determine, by the means of immunohistological procedure, the predominant subsets of lymphocytes (T- and B-cells) in healthy gingiva, gingivitis, and periodontitis tissues. A set of CD antigen specific monoclonal/polyclonal antibodies to detect different cell types infiltrating the gingival tissues were used. These included anti-CD3 (pan T-cell), anti-CD45RO (memory T-cell), anti-CD20 (B-cell), and κ light chain (plasma cells).

The present study showed the presence of both T- and B-cells in healthy and diseased tissues, but in different proportions, which was in accordance with results shown by Modeer *et al.*[22] This could indicate a subclinical inflammation due to constant presence of periodontal pathogens even in clinically healthy gingiva which can then activate the T- and B-cells present in the gingival tissue.

The total T-cell (CD3) infiltrate with respect to B-cell (CD20) infiltrate was higher in the healthy and gingivitis groups, whereas in the periodontitis group B-cells predominated, which is in agreement with the previous studies conducted by Yamazaki and Nakajima *et al.*[6] These cells increased in number with increase in the degree of inflammation as diseased progressed.[23,24]

Upon initial exposure to an antigen a small number of the naive T-cells proliferate and become effector/memory cells which are antigen specific. Memory T-cells express CD45RO. In the healthy and gingivitis groups, T-cells are the predominant type, thus the proportion of memory cells is higher than in periodontitis group.[6]

The priming of the immune cells to prior exposure to plaque bacteria could have resulted in a high proportion of memory to total lymphocytes in the healthy group. Gemmell *et al*, reported that the activation of T- and B-cells occurred within the tissues.[25] Moreover, memory cells in the gingival tissue in health may be retained memory cells following previous activation by periodontal-associated antigens. In diseased tissue the large majority of T-cells being CD45RO+ memory cells, suggests that activated cells in these lesions are re-stimulated naive cells.[26] The increase in CD45RO:CD3 ratio as disease progressed indicates a decrease in the proportion CD3 cells with respect to memory cells in diseased tissue.

The immune cell polarization in the gingival tissue can vary from individual to individual or from community to community as the cell polarization can be determined by several factors such as the pathogen, pathogen recognition receptor on the APCs, APC subsets, microenvironment, and cytokines released by T-cells and other cells in the vicinity.[27]

B-cells serve as a well-controlled part of the adaptive host response and act on systems regulated by T-cells.[11] Depending on the type of T-cell phenotype activated, B-cells can play an important role in humoral immunity. Circulating, naive B-cells (CD20) recognize antigens without the help of APCs, resulting in clonal expansion of sensitized B-cells. For differentitiation and class switching to occur it requires direct contact with memory T-cells.[28] The proportion of B-cell infiltration with respect to the T-cell in the present study was greater in the periodontitis group as suggested by Gemmell *et al.*[29]

Pathogenic bacteria activate B-cells polyclonally in the presence of T-cells into plasma cells.[8,30,31] Polyclonal activation of B-cell by pathogenic bacteria results in the development of antibodies with low avidity, which results in inefficient elimination of bacteria,[11] thus, resulting in persistence of the bacteria which in turn could cause tissue destruction. Jully *et al,*[32] reported that progressive periodontitis was characterized by a B-cell or plasma cell lesion, which was in agreement with the present study. This may be due to hyperactivity influenced by an imbalance in regulatory T-lymphocytes which dominated the periodontal lesion.[32,33] Takahashi *et al,*[13] reported that activated B-cell or plasma cell produced immunoglobulins, with the light chain κ of the immunoglobulin being predominant in periodontitis lesions. Hence, κ light chain was chosen as a marker for activated B-cell or plasma cell.

The ratio of plasma cell: B-cell in our study was low in the healthy and gingivitis groups, indicating that only few B-cells have been converted into plasma cells. These findings are consistent and in agreement with the previous reports by Jully *et al* and Richard *et al.*[32,33] The higher percentage of B-cell infiltration in the periodontitis group compared to other groups and a high ratio of plasma cell:B-cell represents an active infection which has initiated the adaptive immune response resulting in activation of B-cells and increased conversion of B-cells into immunoglobulin-producing plasma cells. Similar findings have been reported by Page and Schroeder, and Ogawa *et al.*[34,35]

The results of this study suggest that it is not only the level of the B- and T-lymphocytes in the gingiva that is important but also their state of activation. The state of activation could decide the cytokine profile that is generated by these cells which thereby regulate disease activity.

Further studies aimed at clarifying the innate immune responses and their relation to memory T-cells could result in better understanding of etiopathogenesis of periodontal disease.
